# Biodegradation of a Magnesium Alloy Fixation Screw Used in a Guided Bone Regeneration Model in Beagle Dogs

**DOI:** 10.3390/ma15124111

**Published:** 2022-06-09

**Authors:** Patrick Rider, Željka Perić Kačarević, Akiva Elad, Daniel Rothamel, Gerrit Sauer, Fabien Bornert, Peter Windisch, Dávid Hangyási, Balint Molnar, Bernhard Hesse, Michel Assad, Frank Witte

**Affiliations:** 1 Department of Prosthodontics, Geriatric Dentistry and Craniomandibular Disorders, Charité–Universitätsmedizin Berlin, Aßmannshauser Straße 4–6, 14197 Berlin, Germany; patrick.rider@botiss.com (P.R.); zpkacarevic@fdmz.hr (Ž.P.K.); 2 Botiss Biomaterials AG, Ullsteinstrasse 108, 12109 Berlin, Germany; a.elad@hotmail.com; 3 Department of Anatomy Histology, Embryology, Pathology Anatomy and Pathology Histology, Faculty of Dental Medicine and Health, University of Osijek, 31000 Osijek, Croatia; 4 CMF Surgery, Johannes BLA Hospital, 41239 Mönchengladbach, Germany; daniel.rothamel@mg.johanniter-kliniken.de (D.R.); gerritsauer@me.com (G.S.); 5 Faculté de Chirurgie Dentaire de Strasbourg, Université de Strasbourg, 8 Rue Sainte-Elisabeth, 67000 Strasbourg, France; bornertfabien@gmail.com; 6 Department of Periodontology, Semmelweis University, 1769 Budapest, Hungary; peter.windisch@gmail.com (P.W.); sosefelejtemel@gmail.com (D.H.); molbal81@gmail.com (B.M.); 7 Xploraytion GmbH, Bismarkstrasse 11, 10625 Berlin, Germany; hesse@xploraytion.com; 8 Medical Device Preclinical Services, Charles River Laboratories, 1635 Lionel-Bertrand Blvd., Boisbriand, QC J7H 1N8, Canada; michel.assad@crl.com

**Keywords:** magnesium fixation screws, beagle dog model GBR, healing, degradation, micro-CT

## Abstract

Nowadays, the most commonly used fixation systems are non-resorbable, but new resorbable magnesium alloy fixation screws have been introduced recently. Therefore, the aim of this study was to compare the magnesium fixation screw and the commonly used non-resorbable titanium screw in an animal model. Four 3-wall defect sites were covered with collagen membranes in the mandible of twenty beagle dogs (two sites on the left and two on the right). Each membrane was fixed with either four magnesium screws or four titanium screws. Post-operative follow-up revealed the expected observations such as transient inflammation and pain. Both groups showed a good healing response, with no differences between groups. Micro-CT analysis showed no significant difference between groups in terms of BV/TV or soft tissue volume. The void volume in the magnesium fixation screw group continued to decrease on average between the different timepoints, but not significantly. Furthermore, a gradual progression of the degradation process of the magnesium screws was observed in the same group. Magnesium screws and titanium screws showed equal performance in tissue regeneration according to GBR principles. An additional advantage of magnesium screws is their resorbable nature, which eliminates the need for a second surgical step to remove the screws.

## 1. Introduction

In the surgical technique of guided bone regeneration (GBR), barrier membranes are used to seclude bony defect sites from the overlying soft connective tissues of the gingiva [1,2]. To prevent migration of the membrane and retain its position over the defect, membranes are often fixated to the underlying bone using various fixation systems such as pins, screws, or tacks. Fixating the membrane also prevents the transfer of micromovements to the augmentation site, which are known to cause the formation of fibrous tissue and prevent bone growth [3,4]. Additionally, the fixation of barrier membranes is associated with improved vertical bone gains and a more rapid formation of an organized bone structure [3,5]. Currently, fixation screws are commonly non-resorbable and made from either titanium or stainless steel. The disadvantage of using these screws include the potential for palpability, intraoral exposure, passive migration, and the distortion of magnetic resonance images [6,7,8]. Due to these issues, the screws are often removed despite having a high stability within the body [9]. It has previously been described that the ideal fixation system would be completely resorbable, provide adequate mechanical fixation for the duration that it is required, and be completely replaced by bone [10]. Benefits of using a resorbable device include the potential to reduce patient discomfort and costs, as well as the duration of therapy [11]. Although non-resorbable fixation devices provide a secure fixation until they are removed, it was suggested by Urban et al. that membrane fixation is only required during the initial formation of a bone matrix within the early weeks of healing [12]. Therefore, this should be a minimum requirement for any resorbable fixation system.

We have previously reported on a resorbable magnesium fixation screw to be used for securing barrier membranes [13]. Magnesium is a biodegradable metal that will be completely resorbed once implanted in the body [14,15]. It has mechanical properties more similar to that of bone than other metals used for fixation screws [16]. In radiographic images, magnesium metal screws have been shown to have a good visibility and produce fewer artifacts than alternative metallic screws, which could be beneficial during the post-operative follow-up [17,18,19]. As magnesium degrades, its metallic structure is transformed from metallic magnesium into magnesium salts, which are subsequently resorbed by the body [20,21]. In addition to the creation of magnesium salts, as the metallic structure degrades it releases hydrogen gas, which can lead to the formation of gas cavities around the implant. However, previous studies using magnesium alloy screws in orthopedics for bone fixation have demonstrated that if gas cavities are present, they will spontaneously resolve once the metallic phase has degraded and will not have a negative effect on bone formation [21,22,23,24,25,26,27,28]. In this current study, we investigate the functionality of the newly developed magnesium fixation screw in a canine mandibular defect model. Collagen barrier membranes were secured over filled, 3-wall defects, at healed extraction sites using either the magnesium fixation screw or a commonly used non-resorbable titanium screw.

## 2. Materials and Methods

### 2.1. Test Item

The test item for this investigation is a biodegradable fixation screw (NOVAMag^®^ fixation screw XS, botiss biomaterials GmbH, Berlin, Germany) that is produced at biotrics bioimplants AG (Berlin, Germany) from the magnesium alloy, WZM211. The screws have a shaft diameter of 1.0 mm, a length of 3.5 mm, and an average weight of 17 mg. The screw has a magnesium fluoride surface to postpone the onset of degradation and maintain a sufficient retention of the barrier membrane.

The head of the screw extends into a triangular prism shape that is referred to as the “drive”. Following a standard insertion protocol as outlined by the manufacturer, the drive is used for transferring torque to the screw during its insertion. Once the screw is seated, the drive is either sheared off or removed using a pair of pliers.

### 2.2. Animals and Anesthesia

In total, 20 adult male beagle dogs (*Canis familiaris*) were used in this study, performed at the Charles River Laboratories, Montreal, ULC. This investigatory study was approved by the testing facility’s Institutional Animal Care and Use Committee (IACUC). The testing facility is also accredited by the Association for the Assessment and Accreditation of Laboratory Animal Care (AAALAC) and the Canadian Council on Animal Care (CCAC). Cohorts of six animals were assigned to 1-week, 8-week, and 16-week timepoints. The remaining two animals were available as spares should there be any morbidity or mortality associated with the investigation. As these spare animals were not needed, they were transferred to a 52-week cohort.

The study required two surgeries, one for teeth extraction (preparatory) and one for the GBR surgery (experimental). Prior to both surgeries, the animals underwent general anesthesia using an injection composed of a mix of Buprenorphine, Acepromazine, and Glycopyrrolate administered intramuscularly. Anesthesia induction for tracheal intubation was achieved with Propofol injected intravenously via a catheter in a vessel of the left or right cephalic or saphenous vein. Upon induction with anesthesia, the subject animal was intubated and supported with mechanical ventilation. Isoflurane in oxygen was administered to maintain a surgical plane of anesthesia and Propofol was injected intravenously as needed to improve the efficacy of the anesthesia.

To achieve local anesthesia for tooth extraction and implantation procedures, as well as manage pain after surgery, 0.8–1.2 mL of Lidocaine mixed with Epinephrine 1:50,000 was administered in each side of the lower jaw. For tooth extraction surgeries, local anesthesia was also administered in each side of the upper jaw.

### 2.3. Surgery

The procedure was performed in two phases: a preparatory and an experimental phase. The preparatory phase involved the surgical extraction of four teeth on each side of the jaw, from the mandibular second premolar to the first molar. The corresponding teeth on the upper jaw were also extracted. Teeth extraction was followed by wound closure and suturing of the upper jaw, whilst the lower jaw remained open during a healing period of 12 ± 2 weeks. Daily oral cavity flushing was performed for 10–14 days post extraction. Sutures were removed from the upper jaw after 2 ± 1 weeks.

**Figure 1:**
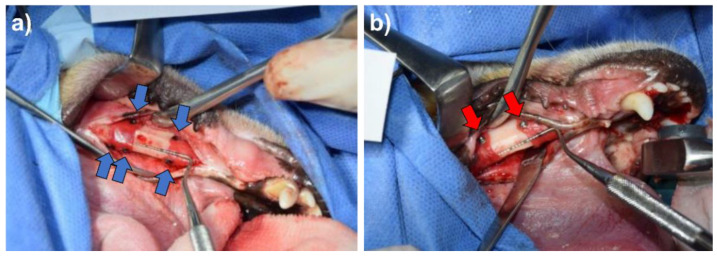
Representative surgical photos for the implantation of (**a**) the magnesium fixation screw and (**b**) the titanium fixation screw, when used to fixate a collagen membrane in a GBR procedure in the lower left jaw in beagle dogs. In both images, two treatment sites are partially visible. The magnesium screws (indicated by the blue arrows) appear black due to their magnesium fluoride surface, whereas the titanium screws appear silver shiny (indicated by the red arrows).

For the experimental phase surgery, two independent 3-wall bone defects were created on each side of the lower jaw. The defects were filled with a bone substitute material (Bio-Oss^®^, Geistlich) and covered with a collagen membrane (Bio-Gide^®^, Geistlich). The collagen membrane was fixated with either 4 magnesium screws or with 4 titanium screws (1.5 mm × 3 mm ProFix titanium screws, Osteogenics), 2 on the buccal side and 2 on the lingual side, followed by wound closure with sutures. Each animal received 4 implanted defect sites, 2 treated with the control titanium screw and 2 with the magnesium screw. Representative photos of the fixation screws after insertion are shown in Figure 1.

Efforts were made by the surgeon to ensure balanced sample size and anatomic distribution for each arm using a non-random design for implantation. Allocation of the magnesium screw or titanium screw was split between the left and right side of the mandible and alternated between dogs. Daily oral cavity flushing was performed for 10–14 days post-implantation and sutures were removed 2 ± 1 weeks post-implantation.

Upon euthanasia, performed using a lethal injection of Pentobarbital (240 mg/mL; 2 mL/4.5 kg), hemimandibles were extracted and stored individually in 100% ethanol and kept refrigerated between 4–8 °C.

### 2.4. Veterinarian Intervention and Care

For the duration of the study, the animals were monitored and observed (cage side observation) at least twice a day by a trained professional. The animals’ health status was followed up by a veterinarian team, as necessary. Post-operative examinations carried out by the veterinarian team were performed under anesthesia using Isoflurane. Scheduled examinations post-implantation occurred three times in the first week (day 1, 3, and 7), once a week for the following three weeks (approximately day 14, 21, and 28), and thereafter once every two weeks (approximately day 42, 56, 70, 84, and 90), or until the day of scheduled sacrifice. The animals were weighed prior to the teeth extraction surgery, the implantation surgery, and sacrifice, as wells as during veterinarian follow-ups.

### 2.5. Micro-CT Collection and Reconstruction

Prior to histological processing, the defects in the explanted hemimandibles were scanned using a Nikon XTH 225 ST Micro-CT scanner [28]. µCT data were then used to reconstruct a 3D image of each defect containing 4 screws (where possible), which consisted of 16-bit volume data at 10 μm isotropic voxel size. To evaluate the regenerative progress for each defect, the new bone volume/total defect volume (BV/TV) and soft tissue volume were measured. Additionally, the magnesium screw treatment group also had the defect void volume measured to evaluate how the hydrogen gas released from the degrading screw influenced the tissue regeneration within the defect site.

**Figure 2:**
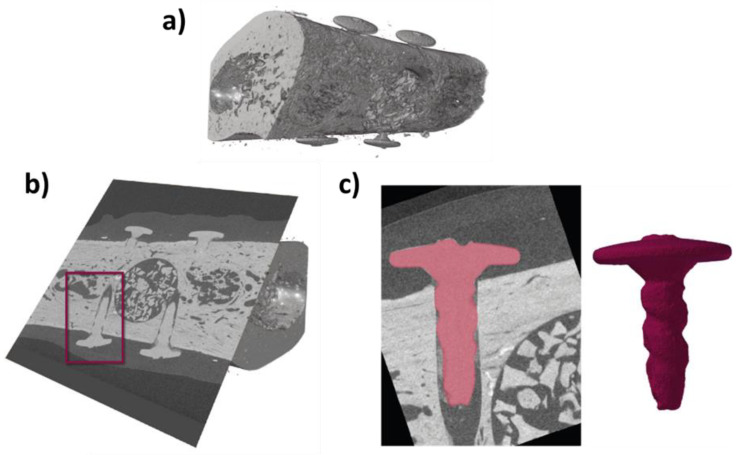
Demonstration of the workflow to obtain the volume and surface area values for each of the implanted screws (3.5 mm in length). µCT scans were taken from the one-week timepoint and show that the magnesium fixations screws remain largely intact. (**a**) µCT of defect; (**b**) Location of the magnesium fixation screws identified; (**c**) Extraction of screw volumes after alignment and segmentation.

To analyze the degradation of the magnesium fixation screw, its volume and surface area were measured. In order to quantify the morphology of the magnesium screw, it had to be segmented within the CT volume. Initially, each implant was virtually cut-out from the reconstructed 3D scans using the software, ImageJ (NIH, Stapleton, NY, USA). The data were then loaded into AVIZO software (Thermo Fisher Scientific, Waltham, MA, USA) and the metallic remnants of the implant were segmented using the Segmentation toolbox of AVIZO. Segmentation was achieved by combining manual segmentation steps together with region growing approaches. Using this technique, the remnant magnesium metal could be differentiated from magnesium salts that are formed during the degradation process. A visual representation of the workflow is demonstrated in Figure 2.

The segmented volumes were then loaded into MatLab (MATLAB and Statistics Toolbox Release 2018b, The MathWorks, Inc., Natick, MA, USA) and every screw analyzed for its volume and surface as well as its surface-area-to-volume ratio.

### 2.6. Statistical Analysis

Statistical analysis was performed by grouping the implants according to their material and implant duration. To compare the regenerative progress between each of the treatment groups, unpaired *t*-tests were performed to identify statistically significant differences for the parameters BV/BT and soft tissue volume between the tested groups at each timepoint. Furthermore, unpaired *t*-tests were used to identify statistically significant differences between sequential timepoints for soft tissue volume and the ratio BV/BT. To analyze the progression of degradation of the magnesium fixation screw, unpaired *t*-tests were used between sequential timepoints for the void volume, magnesium metal surface area, and magnesium metal volume. Statistical analysis was performed using GraphPad Prism 8.1.2 Software.

## 3. Results

### 3.1. Post-Surgical Follow-Up

During the initial 2-week post-surgery period (both the preparatory and experimental surgeries), the expected post-surgical observations were made: acute inflammation and evidence of pain (observed via low food intake, swelling of surgical site and reduced feces output). Post-implantation, both treatment groups demonstrated a good healing response and evolution and there was no noticeable difference between each group. Over the duration of the entire study, animal weights remained within the expected range for beagle dogs having undergone this type of surgery. All animals survived until their scheduled euthanasia.

Post-implantation monitoring reported a few instances of moderate swelling that was equally as likely to occur in either treatment group. Such findings were frequent during the first week post-implantation but less common as the study progressed, with only six animals having issues at later timepoints. At the last scheduled follow-up, all the sites had either healed or showed a good healing progression that was consistent with the elapsed time since implantation. No observations made by the veterinarian that were related to the surgery warranted additional follow-up or treatment beyond that already established in the schedule of the protocol. Almost all of the treated sites for both types of fixation screws reported slight to moderate swelling during the first week post-implantation that subsequently resolved. However, of the 40 treatment sites for each group, 8 sites from the magnesium group and 1 site from the titanium group reported swelling at later timepoints. Observations of swelling at later timepoints in the magnesium fixation screw group mostly occurred around 21 to 28 days post-implantation. In some cases, the swelling was reoccurring; for instance, one magnesium treated site had slight swelling at days 28, 42 and 84, but nothing reported for follow-ups performed on days 56 and 70. At 42 days post-implantation, one dog developed a soft mass approximately 1 cm in diameter at a site treated with the magnesium fixation screw. The soft mass was subsequently drained, and later veterinarian reports stated that a slight swelling and redness remaining at the site. In all other sites that reported swelling, swelling occurred without redness of the overlying tissue of the defect site. At 90 days post-implantation, one site in the titanium screw treatment group was reported to have visible screws through the soft tissue.

Additional documented observations such as low body scoring or appetite, abnormal color or consistency of stools, low feces output, otitis, skin lesions or pododermatitis, and eye discharges, amongst others, were noted for some animals and required special follow-up by veterinary services. However, none of these observations were related to the magnesium or titanium screw, nor had a significant impact on the overall health of the dogs.

### 3.2. Micro-CT Results

**Figure 3:**
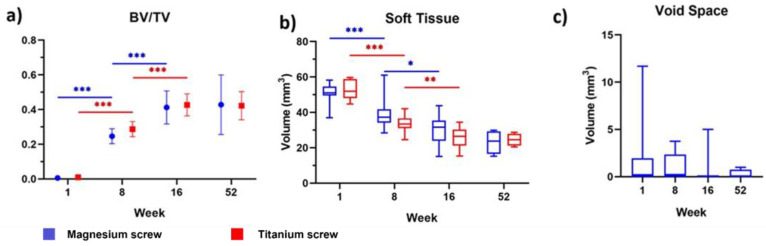
µCT measured values of (**a**) new bone volume/total defect volume, (**b**) soft tissue volume, and (**c**) total void (gas cavity) volume in the defect. Results shown are for defects treated with a bovine bone graft and a collagen membrane fixated with either magnesium (blue) or titanium (red) fixation screws. At each timepoint in (**a**,**b**), there are no significant differences between each treatment group. In (**c**), there are no significant differences between each sequential timepoint. Error bars show the standard deviation in (**a**) and the range in (**b**,**c**). *p* values are represented on the figures as follows: *p* < 0.05: *, *p* < 0.01: **, and *p* < 0.001: ***.

Analysis of the defect sites showed no differences regarding the BV/TV or soft tissue volume between the magnesium and titanium fixation screw groups at every timepoint of the study. The ratio of BV/TV significantly increased at each sequential timepoint between week 1 and 16 for each group (*p* < 0.001), but did not significantly change between week 16 and 52 (Figure 3a). Soft tissue volume also remained non-significantly different between the treatment groups at each timepoint. Soft tissue volume decreased significantly between week 1 and 8 (*p* < 0.001 for both treatment groups), as well as week 8 and 16 (*p* < 0.05 for magnesium and *p* < 0.01 for titanium) (Figure 3b). Between week 16 and 52, there was no significant difference in soft tissue volume for either treatment group. Void volume was only measured for the magnesium fixation screw. It was found that the void volume continued to decrease on average between each timepoint (1 week: 1.98 ± 3.53 mm^3^; 8 weeks: 0.98 ± 1.50 mm^3^; 16 weeks: 0.52 ± 1.45 mm^3^; 0.26 ± 0.50 mm^3^), however, non-significantly (Figure 3c). The magnesium metal of the screw and its fragments could be located and successfully segmented at 1, 8, and 16 weeks. At the 52-week timepoint, remnants of three screws were still visible; however, due to the time elapsed since the screws were implanted, it is likely that the metallic phase has completely degraded leaving mineralized tissue in the form of the screw [14]. Micro-CT measurements of the defect sites were successfully performed, the numerical values of which are reported in Table 1.

**Figure 4:**
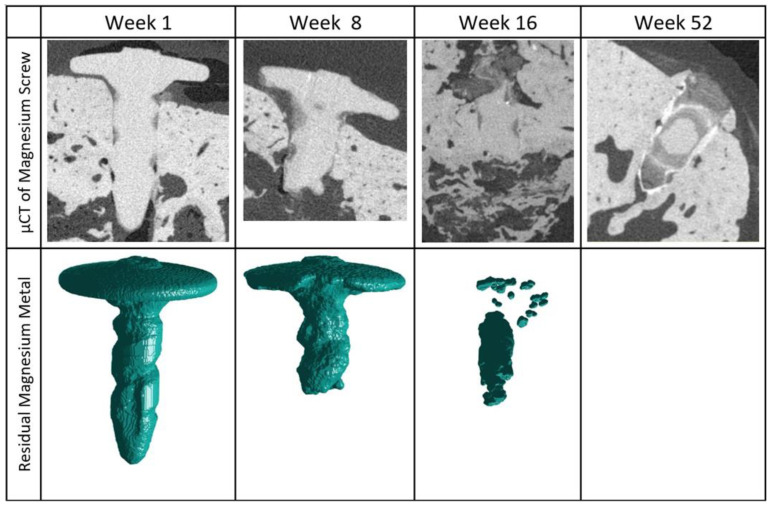
Reconstructed µCT images showing the residual metallic magnesium. The 3D structure of the remaining magnesium metal has been extracted from the µCT data and presented for each timepoint. Due to the small size and few samples with remaining magnesium metal (3 samples with remnants out of 16 samples) at the 52-week timepoint, the residual 3D metallic structure of the screw could not be recreated. Mineralized magnesium degradation products can be seen retaining a partial shape of the degraded fixation screw.

The µCT images show a gradual progression of the degradation process, as demonstrated in Figure 4. One week post-implantation, 4 of the 44 screws had fractured into more than one fragment; however, the rest of the screws remained whole. Each of the four screws had fractured below the head of the screw. In the week 8 cohort, 28 out of 44 screws had fragmented. Visible degradation was present for all of the screws, especially in the region of the screw tip. By 16 weeks, the screws were no longer recognizable due to the advanced stages of degradation. In 11 out of 39 screws, the stem of the screw had fully degraded. After 52 weeks, the majority of the screws appear to be fully corroded in 13 out of 16 cases. Of the three remaining screws, it is unclear if the visible remnants are metallic in structure.

**Figure 5:**
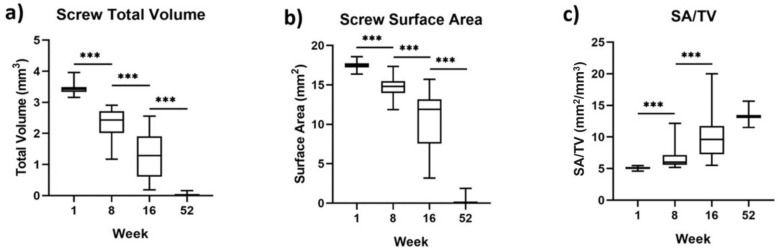
Box and whisker diagrams for the magnesium screw: (**a**) total volume, (**b**) surface area, and (**c**) the ratio of surface area/ total volume, after implantation in a GBR setting in beagle dogs. The whiskers indicate the range of data. *p* values are represented on the figures as follows: *p* < 0.001: ***.

The metallic volume and surface area of the screws were measured at each timepoint, the results of which are shown in Figure 5. At 1 week, the mean total volume of the magnesium screw was 3.43 ± 0.15 mm^3^; at 8 weeks it was 2.35 ± 0.45 mm^3^; and at 16 weeks it was 1.23 ± 0.69 mm^3^. At the 52-week timepoint, the mean total volume of the magnesium screws showed an almost complete degradation and was measured at 0.03 ± 0.06 mm^3^. The corresponding surface area of the screw remnants was 17.49 ± 0.46 mm^2^, 14.76 ± 1.31 mm^2^, 10.31 ± 3.49 mm^2^, and 0.34 ± 0.72 mm^2^ at 1, 8, 16, and 52 weeks, respectively.

## 4. Discussion

In the present study, magnesium fixation screws have been compared to titanium fixations screws to assess their suitability as a membrane fixation system used in GBR treatments. From a safety perspective, all of the animals from both the magnesium and titanium fixation screw groups survived until their scheduled euthanasia. Post-implantation, both treatment groups demonstrated a good healing progression. Although there were more instances of swelling in the magnesium group after the initial week post-implantation, this was not associated with further complications and did not interfere with the healing process. Additionally, neither group indicated the presence of a chronic inflammation reaction such as prolonged redness, swelling, pain, and loss of function, and the dogs maintained a healthy weight for the duration of the study.

Titanium fixation screws are commonly used for membrane fixation in GBR. However, titanium implants are susceptible to intraoral exposure due to their persistent material stiffness causing a mechanical irritation to the mucosal flaps [29]. This was observed in the current study, where one dog had visible titanium screws 90 days post-operation. In contrast, due to the degradative properties of the magnesium screw, none of the screws from this group protruded through the soft tissue.

The purpose of fixating a barrier membrane is to maintain a seclusion of the bony defect and prevent the transfer of micromovements [1,2,3]. Previously, Urban et al. have suggested that membrane fixation is only required during the initial stages of healing while the osteoid is formed [12]. The authors related this suggestion to the resorbable suture Monocryl (clear) 6-0 (Ethicon), which loses approximately half of its mechanical fixation capabilities (tensile strength) after 1 week. In relation to the magnesium screw, it has previously been shown that it retains its mechanical fixation capabilities for over 4 weeks [13], which additionally matches the reported barrier function of a resorbable collagen membrane [30]. In the present study, the ability of the magnesium screw to maintain a secure fixation of a collagen membrane was proven, as evaluation of BV/TV showed no significant difference between the magnesium screw and titanium screw treatment groups.

In both groups, new bone within the defect continued to increase until week 16 and maintained a similar volume at 52 weeks. Both the magnesium and titanium fixation screw groups measured similar proportions of new bone at each timepoint (Table 1). This was also reflected in the volume of soft tissue within each group. Therefore, the µCT analysis of the defect void shows that the progression of tissue regeneration within the defect remained independent of the fixation screw used. Hence, the magnesium fixation screw maintained a secure fixation of the collagen membrane whilst it provided a barrier function during the initial stages of healing.

During the degradation process of magnesium metal, hydrogen gas is released into the surrounding tissue [22]. Due to this known phenomenon, the magnesium screw group additionally had void space measurements made using µCT. The largest void space volume was measured at the 1-week timepoint; however, the void spaces continued to reduce in volume between each of the following timepoints. The initial volume of the void space (1.98 ± 3.53 mm^3^) accounted for approximately 2.5% of the entire defect size (80.95 ± 4.89 mm^3^). Due to the low volume of gas produced and the good tissue diffusion of hydrogen gas [31], the risk of the gas cavities becoming large enough to cause wound dehiscence is low.

Other studies have reported the presence of void spaces around magnesium implants that resolve themselves after the metallic magnesium has degraded, without negatively affecting new bone formation [23,24,25,26,28]. This was also shown in our study, where despite the early formation of void spaces at the 1-week timepoint, the void space continually reduced in volume, whilst equivalent levels of new bone formation were measured for both the magnesium and titanium fixation screw groups at every timepoint.

Signs of acute inflammation such as swelling during healing are an expected potential outcome of GBR surgery and these signs were observed at both magnesium-screw-treated sites and titanium-screw-treated sites. Slightly increased swelling is expected in the magnesium group, due to the induced foreign body reaction caused by the implantation of a resorbable material [32]. In the sites treated with the magnesium screw, this outcome can potentially be explained by the perfusion of magnesium ions into the soft tissue after the degradation of the magnesium screw [31]. Similar observations and complications of biodegradable magnesium screws occurred for soft tissue complications in a clinical retrospective study [33]. However, this was also shown to be a short-term tissue reaction.

The presence of hydrogen gas has also previously been linked to inflammatory reactions [28,33,34]. This could have caused the swelling reported at later timepoints for eight of the magnesium-treated sites, which mostly occurred between 4 and 8 weeks post-implantation. According the µCT data on the volume of the screw, between week 1 and 16 was when the screw was undergoing its most significant degradation and lost its largest volume, hence during this period, it would have released the largest volume of hydrogen gas. Most instances of swelling reported after the first week were only reported once by the veterinarian, which indicates that the swelling was short lived due to the regularity of scheduled veterinarian examinations.

At 52 weeks, the µCT data appear to show screw debris and a remaining outline of the magnesium screw. Due to limitations of the µCT and subsequent analysis technique it was not possible to determine if any of the remnants were metallic in structure. Other studies reporting the clinical application of magnesium alloy orthopedic screws have also found a persistent outline of the magnesium screw long after the screw is expected to have fully corroded [14]. As these studies are clinical, there has been no further analysis made on the observations in the form of bone biopsies; however, Seitz et al. hypothesize that the remnants most likely correspond to mineralized tissue.

## 5. Conclusions

A resorbable magnesium fixation screw has been demonstrated as a viable fixation system for securing barrier membranes during GBR treatments. Compared to titanium screws for fixating collagen barrier membranes over filled defects in beagle dogs, no significant differences were found between each group for the tissue regeneration within the defect. Therefore, it can be concluded that the magnesium fixation screw maintained the position of the barrier membrane, similarly to titanium screws, enabling a seclusion of the overlying gingival tissues whilst new bone was formed. Additionally, the magnesium metal was observed to fully degrade over a period of 52 weeks, and in some instances, its shape was preserved by a presumed mineralized tissue. As the magnesium fixation screw is completely resorbable they do not need to be extracted during a second surgical procedure, which is often performed for the titanium fixation screws. Therefore, the magnesium fixation screw has been demonstrated to offer a secure fixation of barrier membranes during the necessary healing phase, with the additional benefit of being completely resorbable.

## Figures and Tables

**Table 1:** µCT volume measurements for treated GBR defects covered with a collagen barrier membrane that is fixated with either magnesium or titanium fixation screws.

Week	Fixation Screw	No. of Treated Defects	Volume				
			Total Defect (TV) (mm^3^)	New Bone (BV) (mm^3^)	Soft Tissue (mm^3^)	BV/TV	Void Space (mm^3^)
1	Magnesium	12	81.0 ± 5	0.5 ± 0.5	51 ± 5.5	0.01 ± 0.01	2 ± 3.5
	Titanium	12	84.0 ± 8	1 ± 1	52.5 ± 5.5	0.01 ± 0.01	/
8	Magnesium	12	73 ± 6.5	18 ± 3	39 ± 8	0.25 ± 0.04	1 ± 1.50
	Titanium	12	71.5 ± 5	20.5 ± 3	33.5 ± 4.5	0.29 ± 0.04	/
16	Magnesium	12	76.5 ± 8	31.5 ± 8.5	30 ± 8	0.41 ± 0.10	0.5 ± 1.5
	Titanium	12	72 ± 8	30.5 ± 3.5	25.5 ± 6	0.43 ± 0.06	/
52	Magnesium	4	66.50 ± 18	31 ± 19	23 ± 6.5	0.43 ± 0.17	0 ± 0.5
	Titanium	4	68 ± 9	29 ± 9	24.5 ± 3.5	0.42 ± 0.08	/

## Data Availability

The data presented in this article are available on request from the corresponding author.
